# Geography, Deer, and Host Biodiversity Shape the Pattern of Lyme Disease Emergence in the Thousand Islands Archipelago of Ontario, Canada

**DOI:** 10.1371/journal.pone.0085640

**Published:** 2014-01-09

**Authors:** Lisa Werden, Ian K. Barker, Jeff Bowman, Emily K. Gonzales, Patrick A. Leighton, L. Robbin Lindsay, Claire M. Jardine

**Affiliations:** 1 Department of Pathobiology, Ontario Veterinary College, University of Guelph, Guelph, Ontario, Canada; 2 Parks Canada Agency, Thousand Islands National Park, Mallorytown, Ontario, Canada; 3 Canadian Cooperative Wildlife Health Centre, Guelph, Ontario, Canada; 4 Ontario Ministry of Natural Resources, Peterborough, Ontario, Canada; 5 University of Victoria, Victoria, British Columbia, Canada; 6 Department of Pathology and Microbiology, Faulty of Veterinary Medicine, University of Montréal, Saint-Hyacinthe, Quebec, Canada; 7 Public Health Agency of Canada, Winnipeg, Manitoba, Canada; The Johns Hopkins University School of Medicine, United States of America

## Abstract

In the Thousand Islands region of eastern Ontario, Canada, Lyme disease is emerging as a serious health risk. The factors that influence Lyme disease risk, as measured by the number of blacklegged tick (*Ixodes scapularis*) vectors infected with *Borrelia burgdorferi*, are complex and vary across eastern North America. Despite study sites in the Thousand Islands being in close geographic proximity, host communities differed and both the abundance of ticks and the prevalence of *B. burgdorferi* infection in them varied among sites. Using this archipelago in a natural experiment, we examined the relative importance of various biotic and abiotic factors, including air temperature, vegetation, and host communities on Lyme disease risk in this zone of recent invasion. Deer abundance and temperature at ground level were positively associated with tick abundance, whereas the number of ticks in the environment, the prevalence of *B. burgdorferi* infection, and the number of infected nymphs all decreased with increasing distance from the United States, the presumed source of this new endemic population of ticks. Higher species richness was associated with a lower number of infected nymphs. However, the relative abundance of *Peromyscus leucopus* was an important factor in modulating the effects of species richness such that high biodiversity did not always reduce the number of nymphs or the prevalence of *B. burgdorferi* infection. Our study is one of the first to consider the interaction between the relative abundance of small mammal hosts and species richness in the analysis of the effects of biodiversity on disease risk, providing validation for theoretical models showing both dilution and amplification effects. Insights into the *B. burgdorferi* transmission cycle in this zone of recent invasion will also help in devising management strategies as this important vector-borne disease expands its range in North America.

## Introduction

In eastern North America, Lyme disease is a serious emerging health risk caused by the bacterium *Borrelia burgdorferi*, which is transmitted to humans by the bite of an infected blacklegged tick (*Ixodes scapularis*) [1]. Reducing the risk of Lyme disease, as measured by the number of infected nymphal blacklegged ticks in the environment [2], [3], requires a better understanding of the factors that predict the distribution of *B. burgdorferi* and its vectors.

In addition to the tick vector, the *B. burgdorferi* transmission cycle involves a number of mammalian and avian tick hosts [4]. As the primary host for adult blacklegged ticks, white-tailed deer (*Odocoileus virginianus*) are essential for the establishment and maintenance of endemic *I. scapularis* populations [5], [6]. However, as incompetent reservoirs [7], [8] and hosts mainly for adult ticks, deer are unlikely to have an effect on rates of *B. burgdorferi* infection. The effect of deer abundance on tick populations varies across eastern North America [4], [9]. Positive correlations have been seen on isolated islands and using deer exclosures [10]–[12], while studies in areas open to deer movement found little or no correlation between deer population density and the number of blacklegged ticks [9], [13], [14].

White-footed mice (*Peromyscus leucopus*) are considered to be the most competent reservoir for *B. burgdorferi* (most likely to transmit the pathogen to a tick while it is feeding) [15]–[17]. They are ubiquitous in many areas of eastern North America affected by Lyme disease and have been regarded as the primary hosts for larval and nymphal blacklegged ticks in that region (e.g., [18]–[20]). However, eastern chipmunks (*Tamias striatus*) and shrews (*Blarina brevicauda* and *Sorex cinereus*) are also important competent hosts [16], [17], [21]. Squirrels (*Sciurus carolinensis*, *Tamiasciurus hudsonicus*), voles (*Microtus* spp.), raccoons (*Procyon lotor*), and some ground-foraging birds also provide blood meals to many *I. scapularis* larvae and nymphs, but are less competent reservoirs for *B. burgdorferi* [16], [17], [19], [22]–[24].

Differences in host abundance, competence, and quality (lower quality hosts are more likely to groom off and kill ticks that attempt to feed) can affect the number of ticks, the prevalence of infection in ticks, and thus, human risk [16], [25], [26]. Hence, it has been hypothesized that high species richness, with the associated relatively lower abundance of more competent hosts and higher abundance of less competent and low-quality hosts, may lead to a decrease in human disease risk, as measured by the abundance of infected nymphal ticks [22], [27], [28]. Although several studies have provided evidence for this “dilution effect,” predominantly through modelling, not all have, and the mechanisms behind the effects of host abundance, community composition, and species richness on human risk are still poorly understood [25], [26], [29]–[31].

Environmental conditions, including temperature, relative humidity, canopy cover, and leaf litter, can affect the survival and development of ticks [32]–[34]. Canopy cover and leaf litter reduce extremes in temperature and humidity by providing shade and keeping more moisture at the level of the forest floor, which increases tick survival [32], [33]. Tick development is limited by colder temperatures, which slows questing activity (i.e., searching for a blood meal) and can prolong the interval between moults [33], [35], [36]. Projected climatic warming will likely expand the range of ticks in ecologically suitable areas of southern Canada in the coming decades [1], [37].

Elevated tick numbers often represent established tick populations in which, in the early stages of establishment, the prevalence of *B. burgdorferi* infection may be lower than the prevalence in bird-dispersed adventitious ticks [38]. Over time, however, endemic cycles of *B. burgdorferi* transmission can cause the prevalence of infection in the local tick population to increase and eventually exceed the prevalence in adventitious ticks [7], [39]. Until relatively recently, established *I. scapularis* populations recognized in Ontario have been restricted to localized areas along the north shore of Lake Erie, Lake Ontario, and the St. Lawrence River [40]. However, blacklegged ticks and *B. burgdorferi* are spreading across northeastern North America from original endemic foci in the United States and are increasingly being confirmed as endemic in parts of eastern Canada [41]–[48]. Lyme disease had spread from southeastern New York and Connecticut throughout New York State by the mid-1990s, including to Jefferson and St. Lawrence Counties, [42], [49] and in 2006 populations of blacklegged ticks and *B. burgdorferi* were discovered in the adjacent Thousand Islands region of eastern Ontario (unpublished data).

Despite being distributed within an area about 28 km long and 8 km wide, there is variation among sites in the Thousand Islands in the number of blacklegged tick vectors, the prevalence of infection in those ticks, and the diversity of the small mammal host community [50]. This created a natural experiment to evaluate the factors influencing tick establishment in a zone of recent invasion. We examined the relative importance of proximity to the United States, deer abundance, temperature at ground level, canopy cover, leaf litter, small mammal species richness, number of *I. scapularis* nymphs, and relative abundance of mice in explaining the number of nymphs (NON), the prevalence of *B. burgdorferi* infection in nymphs (NIP), and the number of infected nymphs (NIN) in the heavily-visited Thousand Islands region. Given the diversity of small mammal communities in the Thousand Islands archipelago, we were also interested in whether species richness would affect NON, NIP and NIN differently depending on the relative abundance of mice. This information can guide management to control blacklegged ticks and reduce the risk of Lyme disease in the Thousand Islands as this vector-borne disease system expands its range in North America. Our study is one of the first to consider the interaction between the relative abundance of small mammal hosts and species richness in the analysis of the effects of biodiversity on disease risk, providing validation for theoretical models exploring dilution and amplification effects.

## Materials and Methods

### Study Area

**Figure 1:**
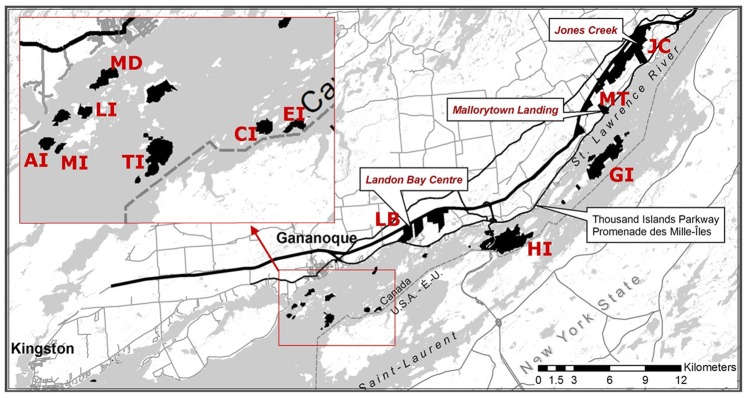
Map of Thousand Islands National Park in eastern Ontario. Two-letter codes identify the 12 study sites on park property. Mainland sites: LB–Landon Bay, MT–Mallorytown, JC–Jones Creek. Island sites: AI–Aubrey, MI–Mermaid, LI–Lindsay, MD–McDonald, TI–Thwartway, CI–Camelot, EI–Endymion, HI–Hill, GI–Grenadier. (Base map courtesy of Parks Canada).

The study area was comprised of 12 sites within Thousand Islands National Park in the Canadian Thousand Islands (44.45320°N, 75.86085°W; Fig. 1). Nine sites were located on islands of different sizes (seven ranged from 1.7 to 40 ha; Grenadier and Hill islands were 406 and 531 ha, respectively) along 28 km of the St. Lawrence River. The remaining three sites were located on the Canadian mainland less than 1 km from the river and no more than 11 km from at least one island study site (Fig. 1). In spring 2009, locations for 121 traps were marked at the intersection of right angle grid lines laid out at 10-m intervals in a 100-m^2^ (one hectare) grid at each study site (except Mermaid Island, which, because of its small size, had only 100 traps and a circular trap layout at 10-m intervals). The same trap locations were used for the duration of the study.

### Drag Sampling

Each of the 12 sites was surveyed for ticks by drag sampling in June, August, and October 2009 and May, June, August, and October 2010 to correspond with periods of peak activity of each tick life stage [51], [52]. A 1-m^2^ flannel sheet was dragged through the vegetation in each one-hectare study plot for a total of two person hours at each sampling session. Drag sampling was done during daylight hours when the temperature was above 4°C and the vegetation was not wet. Researchers attempted to avoid covering the same ground twice while dragging the entire one-hectare plot. Stopwatches were used to ensure drag times were consistent. Sheets were checked every two to five minutes and nymphal ticks were removed. The time taken to check the sheets was not included in the drag time. Ticks were placed in split-top vials in the field, then killed in 99% isopropyl alcohol and stored at −20°C until they were shipped to the Public Health Agency of Canada National Microbiology Laboratory in Winnipeg, where they were stored at −80°C prior to testing.

### Tick Identification and Testing

All nymphs collected by drag sampling in 2009 and 2010 were counted and identified to species using the taxonomic key of Durden and Keirans [53]. Ticks identified as *I. scapularis* were tested for *B. burgdorferi* by polymerase chain reaction (PCR) following the protocols described for *B. burgdorferi* in [54]. Briefly, DNA was extracted from individual nymphs using commercial DNA extraction kits (96-well format DNeasy, QIAGEN, Inc., Toronto, ON, Canada). Amplification of DNA was accomplished using an Applied Biosystem Prism 7900 sequence detector (Applied Biosystems, Carlsbad, CA, USA) using standard thermocyclers and two-percent agarose gels. Samples were considered positive when they produced cycle threshold values <40 with two different primer and probe sets on real-time PCR. Appropriate positive and negative (water) controls were incorporated into every set of PCR reactions. To ensure DNA extraction was successful in ticks, PCR was performed on all tick extracts using primers and a probe that targets the 16S gene of *Ixodes* tick species [54]. If more than 30 nymphs were collected during one sampling session, a minimum of 30 were arbitrarily selected for testing.

### Small Mammal Trapping

Small mammals were trapped and handled in Thousand Islands National Park under a Research and Collection Permit approved by the Parks Canada Agency and in accordance with an Animal Utilization Protocol (09R039) approved by the University of Guelph Animal Care Committee following the guidelines of the Canadian Council on Animal Care.

Sherman live traps (H.B. Sherman Traps, Inc., Tallahassee, FL, USA) were set at marked grid locations at each site for four consecutive nights in June and August. Traps were baited with sunflower seeds and contained polyester or natural cotton bedding. Traps were set in the evening and checked before 08∶00 the following morning. Animals were identified to species (to genus for *Peromyscus* spp.) based on phenotype [55]. In 2009, 30 animals identified as *Peromyscus* sp. were all confirmed as *P. leucopus* by genetic analysis of ear punch samples using methods described by Thompson [56]. All animals subsequently identified as *Peromyscus* were assumed to be white-footed mice. Ticks were collected from all animals as part of a concurrent study and animals were then released at their point of capture (see [50] for details).

The abundance of animals at each site was an index of abundance expressed as the number of animals caught per 100 trap nights each year. The relative abundance of *Peromyscus* population was categorized as “low” when mice made up 50% or less of the small mammal captures at a site, and as “high” when mice were more than 50% of the animals captured. Analyses were conducted using the actual proportions of mice present. Relative mouse abundance was categorized only to aid in visual interpretation of the figures depicting the effects of the interaction between species richness and relative mouse abundance on NON, NIP, and NIN; when depicted as a continuous variable, the effect of mouse abundance was qualitatively similar. Species richness was expressed as the number of small mammal species trapped at each location over the two years of trapping.

### Climate Monitoring

A data logger (HOBO Pro v2, Onset Computer Corporation, Pocasset, MA, USA) positioned one to two cm above the ground near the centre of each one-hectare study site recorded air temperature and humidity at 30-minute intervals from July 2009 to the spring of 2011. Data were collected from the data loggers periodically (approximately every 300 days) and compiled using HOBOware Pro version 3.2.1 (Onset Computer Corporation, Pocasset, MA, USA). The average daily minimum temperatures were calculated for three-month periods (January-March, April-June, July-September, October-December) because ticks are sensitive to extremes in microclimatic variation and climate variables are typically grouped seasonally to coincide with tick activity periods (e.g., [33], [57], [58]).

### Deer Abundance

Relative white-tailed deer abundance was estimated by pellet counts [59]. Each site was surveyed in the fall of 2009 for deer pellets. The number of groups of pellets within a circular survey plot with a 1.7-m radius (9.29 m^2^/100 square feet) around each trap location was recorded and survey plots were cleared of pellets. Each survey plot was assessed for pellet groups again in the spring of 2010. The accumulation of pellets from fall 2009 to spring 2010 was used to calculate the number of pellet groups per hectare, which was used as a relative index of deer abundance at each site.

### Vegetation

Average leaf litter depth at each site was determined in September 2010 by inserting a ruler into the litter layer until bare ground was reached at 11 randomly selected trap locations. The percent canopy cover at each trap location was categorized (<25%, 25–49%, 51–75%, or >75%) and used to calculate the average overall canopy cover for the study site.

### Geography

Distance to the United States was measured in ArcGIS (version 9.3, Redlands, CA, USA) as the shortest straight-line distance to the American mainland from the shore of the island on which the study site was located or from the point on the Canadian shore of the St. Lawrence River closest to the mainland study site. Because some of the sites were located in close proximity to one another and model residuals showed significant autocorrelation by a Moran’s I test, an autocovariate term was used to account for the spatial non-independence of sites, calculated as an inverse distance-weighted function of the response variable in neighboring sites [60]–[62].

### Statistical Analyses

To assess the effects of various factors (Table 1) on tick populations and *B. burgdorferi* infection in ticks (Table 2), an information theoretic approach was used [63]. We used linear regression models fitted to log-transformed tick abundances to model NON and NIN, and logistic regression to model NIP. The importance of various random effects was assessed in each case using the full model containing all fixed effect terms, and an appropriate random effects structure was selected and retained during subsequent model selection [64]. Next, we computed regression models for all possible combinations of fixed effect variables. AIC_c_ (Akaike’s information criterion corrected for small sample size [63]) values were then used to select a 95% confidence set of candidate models for use in inference. We calculated composite parameter estimates and standard errors incorporating model selection variance for all parameters in the confidence set using conventional model averaging [63].

**Table 1:** A description of variables sampled in a study of the distribution of *Ixodes scapularis* and the prevalence of *Borrelia burgdorferi* infection in ticks in the Thousand Islands region of Ontario, Canada, in 2009 and 2010.

Parameter	Description
Temperature	Average daily minimum temperature July-September
Mice	Relative abundance of *Peromyscus leucopus*
Richness	Small mammal species richness
Canopy	Percent canopy cover
Deer	Average number of pellet groups per hectare
Distance	Distance from United States mainland (km)
Leaf litter	Average depth of leaf litter (cm)
Richness*Mice	Interaction between Mice and Richness
Animals	Number of small mammals trapped per 100 trap nights
Number of nymphs	Nymphs caught per 2 person hours of dragging
Autocorrelation	Spatial autocorrelation term [60]–[62]

**Table 2:** Variables examined for associations with the abundance of *Ixodes scapularis* nymphs and the prevalence of *Borrelia burgdorferi* in nymphal ticks in the Thousand Islands region of Ontario, Canada in 2009 and 2010.

Outcome variable	Description	Variable (predicted direction of relationship)
NON	Number of nymphs per two person hours of dragging	Distance to U.S. (−)
		Deer (+)
		Canopy cover (+)
		Leaf litter (+)
		Temperature (+)
		Mice*richness (+/−)
NIP	Prevalence of *B. burgdorferi* infection in nymphs (nymphal infection prevalence)	Distance to U.S. (−)
		Number of animals (−)
		Number of nymphs (+)
		Mice*richness (+/−)
		Autocorrelation (+)
NIN	Number of infected nymphs per two person hours of dragging	Distance to U.S. (−)
		Deer (+)
		Number of nymphs (+)
		Canopy cover (+)
		Mice*richness (+/−)

When selecting a random effects structure using the full model of fixed effects, site and year did not explain additional variation beyond that already captured by the fixed effects and were not retained. However, the inclusion of month as a random effect to control for the variation among seasons improved the NON and NIN models substantially as measured by AICc. To allow comparison of the relative effects of the coefficients, continuous fixed effects variables were standardized to have a mean of zero and standard deviation of one [65]. Variance inflation factors and correlation coefficients were used to check for collinearity among the fixed effect variables and remove variables exhibiting strong collinearity. Statistical analyses were conducted using R version 2.14.0 [66].

## Results

A total of 1354 nymphal *I. scapularis* were collected in 2009 and 2010. Nymphs were found at all 12 sites. Nymphal ticks tested positive for *B. burgdorferi* infection at eight of the 12 sites, with infection prevalence ranging from 12.3 to 29.6%. No infected nymphs were collected at the three mainland sites or Mermaid Island. A total of 771 primary (first-time) captures of eight small mammal species were recorded during 22,895 trap nights in 2009 and 2010 (Table 3). There was a positive correlation between species richness and the overall abundance of small mammals (rho = 0.529, p<0.001). Deer density increased further from the United States (rho = 0.455, p = 0.026), while small mammal species richness decreased (rho = −0.754, p = 0.005).

**Table 3:** Primary captures of animals at 12 sites in the Thousand Islands during trapping sessions in June and August 2009 and 2010 included the white-footed mouse (*Peromyscus leucopus*), short-tailed shrew (*Blarina brevicauda*), eastern chipmunk (*Tamias striatus*), meadow vole (*Microtus pennsylvanicus*), flying squirrel (*Glaucomys* sp.), masked shrew (*Sorex cinereus*), red squirrel (*Tamiasciurus hudsonicus*), and short-tailed weasel (*Mustela erminea*).

Site^a^	Size(hectares)	Trapnights	*Peromyscus*(relative abundance)	*Blarina*	*Tamias*	*Microtus*	*Glaucomys*	*Sorex*	*Tamiasciurus*	*Mustela*	Total	Richness
AI	6.8	1936	1 (50%)	0	1	0	0	0	0	0	2	2
CI	9.4	1935	57 (83%)	0	0	11	0	0	1	0	69	3
EI	5.7	1936	1 (2%)	8	0	37	0	0	0	0	46	3
GI	406.6	1936	105 (90%)	6	4	0	0	0	0	2	117	4
HI	531	1936	70 (62%)	15	23	0	3	0	1	0	112	5
JC	Mainland	1936	71 (55%)	24	25	0	4	4	1	0	129	6
LB	Mainland	1936	19 (50%)	9	10	0	0	0	0	0	38	3
LI	14.4	1936	12 (46%)	10	0	4	0	0	0	0	26	3
MD	15.1	1936	41 (93%)	0	0	3	0	0	0	0	44	2
MI	1.7	1600	2 (14%)	3	0	9	0	0	0	0	14	3
MT	Mainland	1936	44 (44%)	29	22	0	3	1	0	0	99	5
TI	40	1936	73 (97%)	0	0	2	0	0	0	0	75	2
												
Total		22895	496	104	85	66	10	5	3	2	771	8

^a^ AI–Aubrey Island, CI–Camelot Island, EI–Endymion Island, GI–Grenadier Island, HI–Hill Island, JC–Jones Creek, LB–Landon Bay, LI–Lindsay Island, MD–McDonald Island, MI–Mermaid Island, MT–Mallorytown, TI–Thwartway Island.

### Number of Nymphs

**Figure 2:**
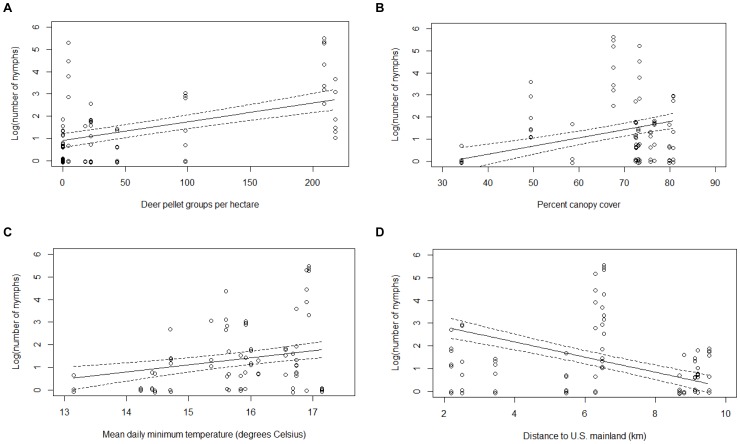
Predicted number of nymphs. Actual data points and predicted number of *Ixodes scapularis* nymphs per two person hours of drag sampling (NON) and standard errors as a function of a) deer abundance, b) percent canopy cover, c) mean daily minimum summer temperature, and d) distance to the United States at various sites in the Thousand Islands. These variables were retained in the best model, which explained 51% of the variance in the number of nymphs.

**Figure 3:**
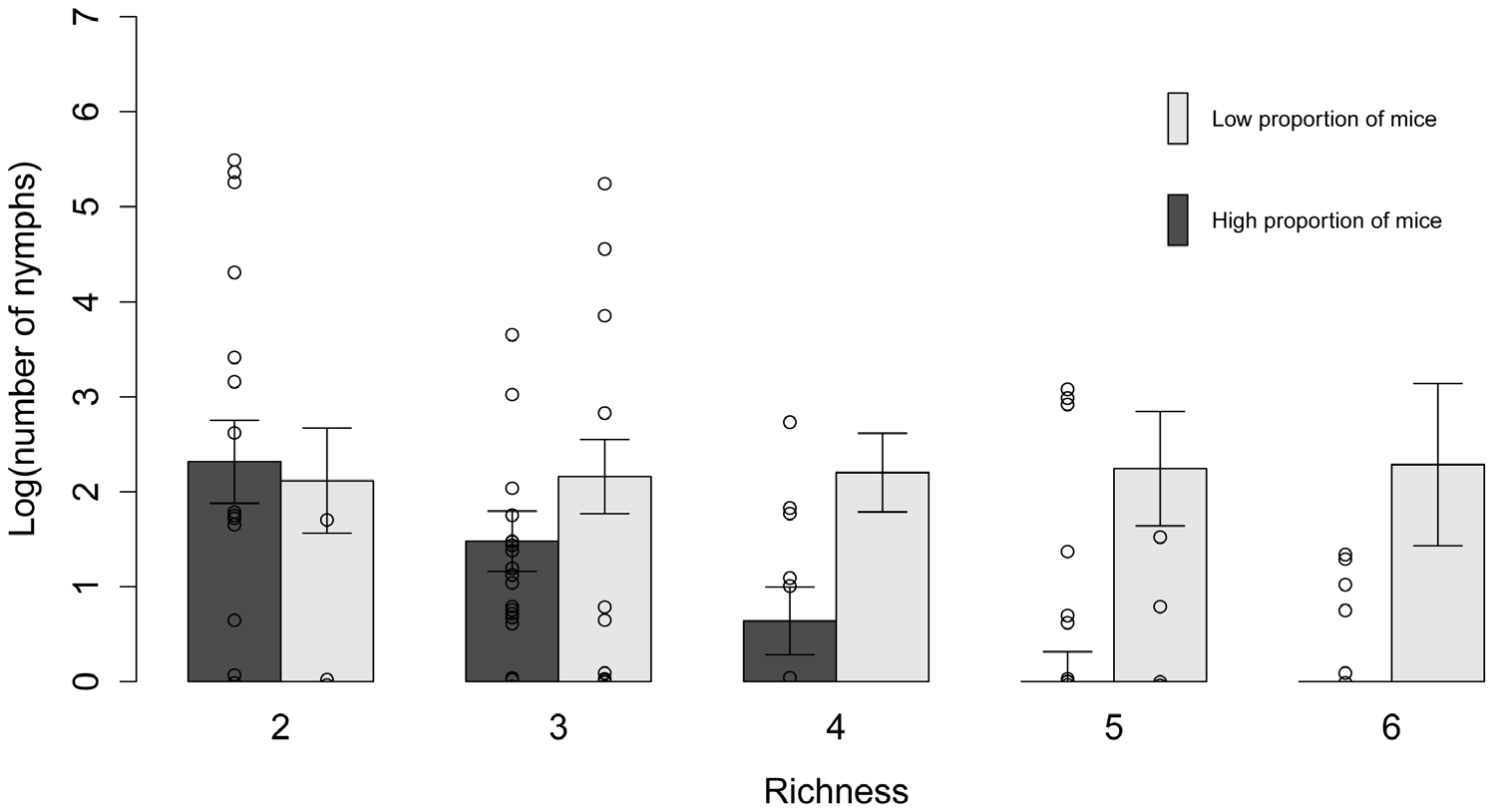
Predicted number of nymphs as a function of small mammal species richness. Actual data points and predicted number of *Ixodes scapularis* nymphs per two person hours of drag sampling (NON) and standard errors as a function of small mammal species richness at low (≤50%) and high (>50%) relative abundance of mice at various sites in the Thousand Islands. The interaction between species richness and the relative abundance of mice was retained in the best model, which explained 51% of the variance in the number of nymphs.

For the models examining the factors affecting the NON, nine of the 255 candidate models were retained in the 95% confidence set, as determined by AIC_c_ values (Table 4). The models in the confidence set individually explained between 29 and 51% of the variation in the data. Distance to the United States and deer abundance appeared in all nine models in the confidence set. Average daily minimum summer temperature, percent canopy cover, and the interaction between small mammal species richness and the relative abundance of mice were also important predictors (Table 5). There was little support for leaf litter depth as an important variable in the models. Deer abundance, canopy cover, and average daily minimum summer temperature were positively correlated with the number of nymphs, and the relationship between the number of nymphs and distance to the United States was negative (Fig. 2). There was an overall negative effect of small mammal species richness on the NON. This effect was driven by sites with mouse-dominated communities and not supported in communities with a low proportion of mice, as demonstrated by an important interaction between the relative abundance of mice and species richness (Fig. 3). The overall abundance of small mammals was not correlated with the number of nymphs (rho = 0.157, p = 0.164).

**Table 4:** 95% confidence set of best ranked linear mixed models selected through all subsets regression analysis to explain the number of *Ixodes scapularis* nymphs (NON)^a^ at various locations in the Thousand Islands in 2009 and 2010.

Model rank	Model terms	Parameters	R^2^	AIC_c_	Δ*_i_*	w*_i_*	Cumulative w*_i_*
1	Temperature+Deer+Mice+Canopy+Distance+Richness*Mice	10	0.51	220.24	0	0.4660	0.4660
2	Temperature+Deer++Mice+Richness+Leaf Litter+Canopy+Distance+Richness*Mice	11	0.54	222.22	1.98	0.1732	0.6391
3	Deer+Mice+Richness+Canopy+Distance+Richness*Mice	9	0.50	222.81	2.57	0.1292	0.7683
4	Deer+Richness+Leaf Litter+Canopy+Distance+Richness*Mice	10	0.40	224.08	3.84	0.0684	0.8367
5	Temperature+Deer+Mice+Richness+Canopy+Distance	9	0.52	224.63	4.39	0.0519	0.8886
6	Temperature+Deer+Mice+Richness+Leaf Litter+Canopy+Distance	10	0.52	226.32	6.08	0.0223	0.9109
7	Deer+Distance+Richness*Mice	8	0.29	226.61	6.37	0.0193	0.9302
8	Temperature+Deer+Distance+Richness*Mice	9	0.42	227.28	7.04	0.0138	0.9440
9	Temperature+Deer+Mice+Richness+Leaf Litter+Distance+Richness*Mice	10	0.51	228.05	7.81	0.0094	0.9534

**Model** **terms:** Temperature = average daily minimum temperature July-September; Deer = average number of pellet groups per hectare; Mice = relative abundance of *Peromyscus leucopus*; Richness = small mammal species richness; Canopy = percent canopy cover; Distance = distance from United States mainland (km); Leaf Litter = average depth of leaf litter (cm); Richness*Mice = interaction between Mice and Richness. Month was included as a random effect.

**Other abbreviations:** R^2^ = proportion of variance explained by the model, adjusted for number of terms and sample size; AIC_c_ = Akaike’s information criterion corrected for sample size; Δ*_i_* = difference in AIC_c_ from top-ranked model; w*_i_* = AIC weight [63].

^a^ Results are for transformed data (log(NON+1)).

**Table 5:** Model-averaged parameter estimates and standard errors based on the 95% confidence set for variables potentially affecting the number of *Ixodes scapularis* nymphs (NON)* at various locations in the Thousand Islands in 2009 and 2010.

			95% confidence interval	
Parameter	Estimate	Standard error	Upper	Lower
Distance	−0.85	0.21	−0.44	−1.27
Richness	−0.76	0.24	−0.30	−1.23
Deer	0.62	0.17	0.95	0.29
Mice	−0. 61	0.18	−0.27	−0.95
Canopy	0.43	0.15	0.73	0.13
Mice*Richness	−0.74	0.27	−0.21	−1.26
Temperature	0.34	0.15	0.64	0.05
Leaf Litter	−0.13	0.15	0.17	−0.43

Results are for transformed data (log(NON+1)).

**Model** **terms:** Temperature = average daily minimum temperature July-September; Deer = average number of pellet groups per hectare; Mice = relative abundance of *Peromyscus leucopus*; Richness = small mammal species richness; Canopy = percent canopy cover; Distance = distance from United States mainland (km); Leaf Litter = average depth of leaf litter (cm); Mice*Richness = interaction between Mice and Richness. Month was included as a random effect.

### Prevalence of *B. burgdorferi* Infection

**Figure 4:**
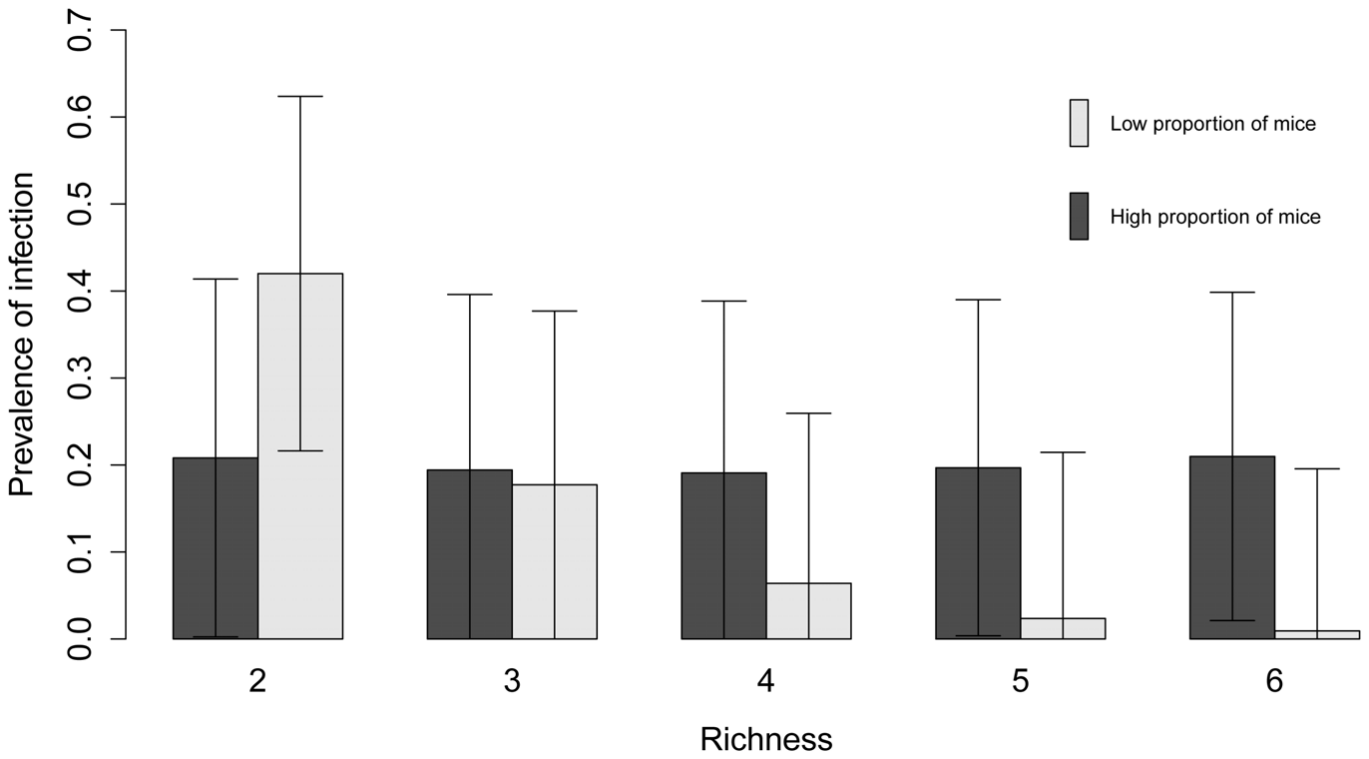
Predicted prevalence of *Borrelia burgdorferi* infection in nymphs as a function of species richness. Predicted prevalence of *Borrelia burgdorferi* infection in *Ixodes scapularis* nymphs (NIP) and standard errors as a function of small mammal species richness at low (≤50%) and high (>50%) relative abundance of mice at various sites in the Thousand Islands. The interaction between species richness and the relative abundance of mice was retained in the best model, which explained 3.2% of the deviance in the prevalence of infection in nymphs.

**Figure 5:**
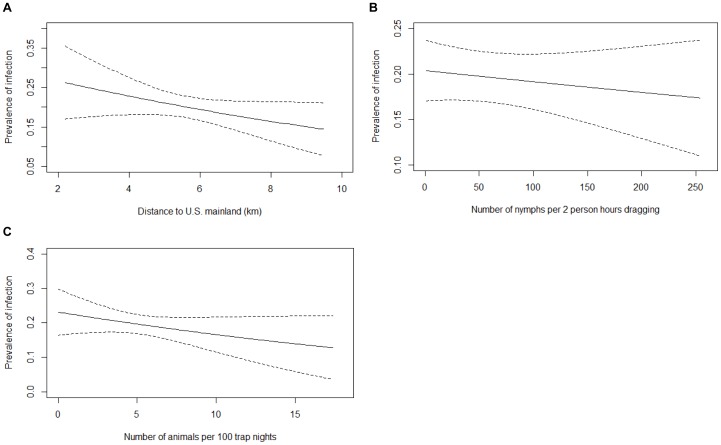
Predicted prevalence of *Borrelia burgdorferi* infection in nymphs. Predicted prevalence of *Borrelia burgdorferi* infection in *Ixodes scapularis* nymphs (NIP) and standard errors as a function of a) distance to the United States, b) number of nymphs, and c) number of small mammals at various sites in the Thousand Islands. These variables were retained in the best model, which explained 3.2% of the deviance in the prevalence of infection in nymphs.

Of the 127 models explored to assess the factors affecting the NIP, four were retained in the 95% confidence set (Table 6); each model explained between 2.8 and 3.2% of the variation in the data. Of the variables considered, the interaction between small mammal species richness and the relative abundance of mice was the most important predictor of the prevalence of infection in nymphal ticks (Table 7). The overall negative effect of small mammal species richness on the NIP was driven by the sites with a low relative abundance of mice (Fig. 4). At sites where the relative abundance of mice was high, greater species richness had little apparent effect on the prevalence of *B. burgdorferi* in nymphs (Fig. 4). The number of nymphs, distance to the United States, and the number of small mammals were other important variables in predicting the *B. burgdorferi* infection prevalence in nymphs; all were weakly negatively correlated with infection prevalence (Fig. 5). The autocorrelation in the prevalence of *B. burgdorferi* infection among sites was not an important factor (Table 7).

**Table 6:** 95% confidence set of best ranked generalized linear models selected through all subsets regression analysis to explain the prevalence of *Borrelia burgdorferi* infection (NIP) in nymphal *Ixodes scapularis* collected at various locations in the Thousand Islands in 2009 and 2010.

Model rank	Model terms	Parameters	D^2^	AIC_c_	Δ*_i_*	w*_i_*	Cumulative w*_i_*
1	Animals+Distance+Nymphs+Richness*Mice	6	0.032	1455.82	0	0.5458	0.5458
2	Animals+Autocorrelation+Distance+Nymphs+Richness*Mice	7	0.032	1457.23	1.41	0.2692	0.8150
3	Distance+Nymphs+Richness*Mice	5	0.028	1459.18	3.36	0.1017	0.9167
4	Autocorrelation+Distance+Nymphs+Richness*Mice	6	0.028	1460.95	5.13	0.0420	0.9587

**Model** **terms:** Animals = number of small mammals trapped per 100 trap nights; Distance = distance from United States mainland (km); Number of nymphs = nymphs caught per 2 person hours of dragging; Richness = small mammal species richness; Mice = relative abundance of *Peromyscus leucopus*; Richness*Mice = interaction between Mice and Richness; Autocorrelation = spatial autocorrelation term [60]–[62].

**Other abbreviations:** D^2^ = proportion of deviance explained by the model, weighted by residual degrees of freedom [60]; AIC_c_ = Akaike’s information criterion corrected for sample size; Δ*_i_* = difference in AIC_c_ from top-ranked model; w*_i_* = AIC weight [63].

**Table 7:** Model-averaged parameter estimates and standard errors based on the 95% confidence set for variables potentially affecting the prevalence of *Borrelia burgdorferi* infection in nymphal *Ixodes scapularis* collected at various locations in the Thousand Islands in 2009 and 2010.

			95% confidence interval	
Parameter	Estimate	Standard error	Upper	Lower
Mice*Richness	0.67	0.20	1.05	0.29
Richness	−0.41	0.13	−0.15	−0.66
Distance	−0.39	0.12	−0.15	−0.64
Number of nymphs	−0.26	0.09	−0.10	−0.43
Mice	0.24	0.15	0.53	−0.05
Animals	−0.25	0.11	−0.04	−0.46
Autocorrelation	0.12	0.16	0.43	−0.20

**Model** **terms:** Animals = number of small mammals trapped per 100 trap nights; Distance = distance from United States mainland (km); Number of nymphs = nymphs caught per 2 person hours of dragging; Richness = small mammal species richness; Mice = relative abundance of *Peromyscus leucopus*; Mice*Richness = interaction between Mice and Richness; Autocorrelation = spatial autocorrelation term [61].

### Number of Infected Nymphs

**Figure 6:**
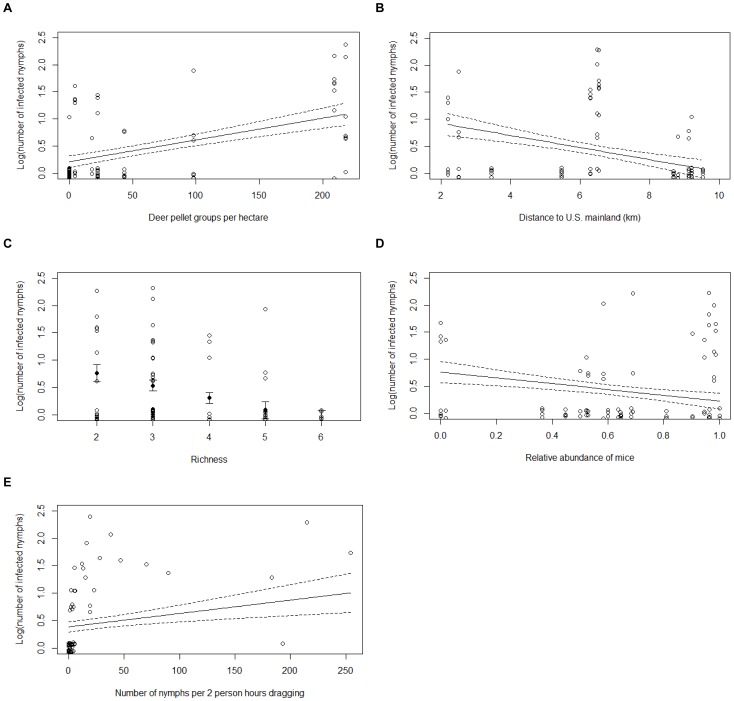
Predicted number of infected *Ixodes scapularis* nymphs. Actual data points and predicted number of *Ixodes scapularis* nymphs infected with *Borrelia burgdorferi* per two person hours of drag sampling (NIN) and standard errors as a function of a) deer abundance, b) distance to the United States, c) small mammal species richness, d) relative abundance of mice, and e) number of nymphs at various sites in the Thousand Islands. These variables were retained in the best model, which explained 45% of the variance in the number of infected nymphs.

**Figure 7:**
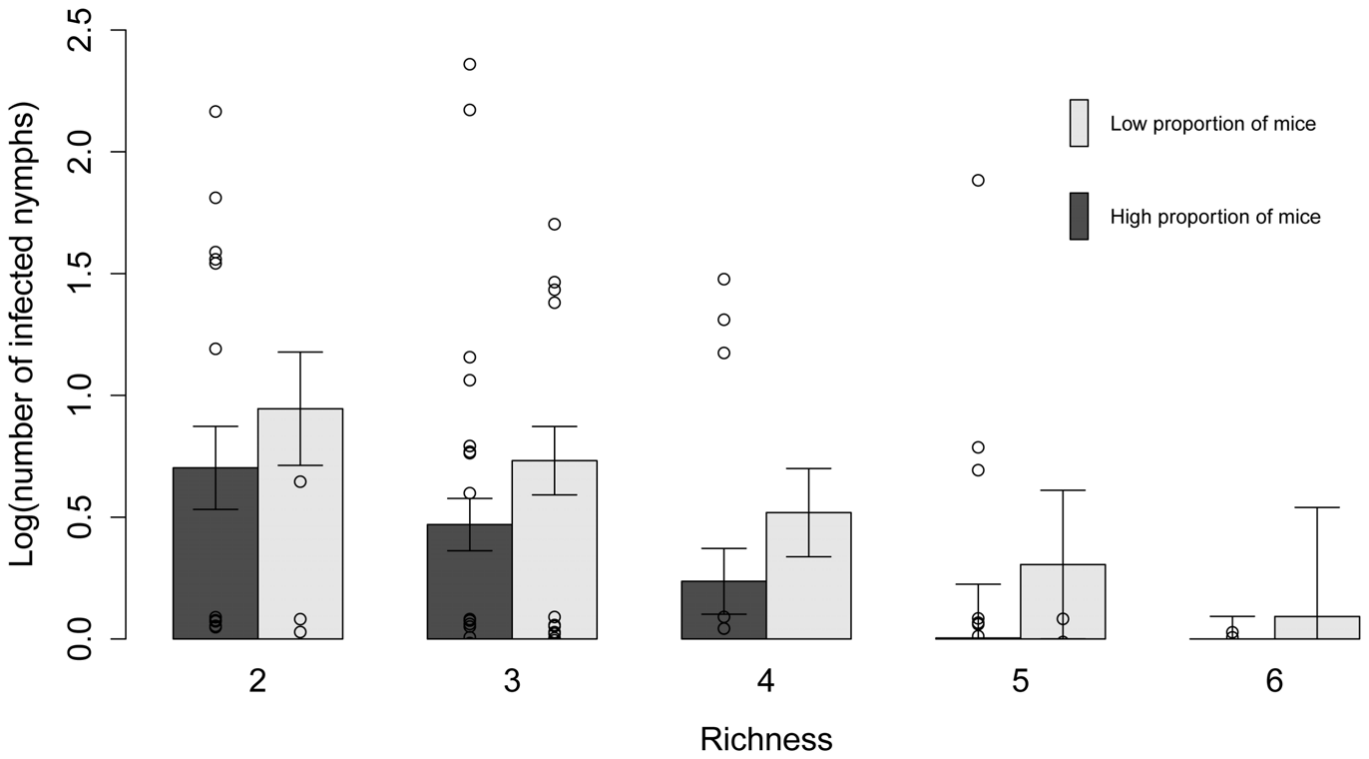
Predicted number of infected nymphs as a function of small mammal species richness. Actual data points and predicted number of *Ixodes scapularis* nymphs infected with *Borrelia burgdorferi* per two person hours of drag sampling (NIN) and standard errors as a function of small mammal species richness at low (≤50%) and high (>50%) relative abundance of mice at various sites in the Thousand Islands. The interaction between species richness and the relative abundance of mice was not retained in the best model.

To examine the factors affecting the number of infected nymphs (NIN), 127 models were considered; 19 were included in the 95% confidence set (Table 8). Between 34 and 45% of the variation in the data was explained by the confidence set of models. Deer abundance, distance to the United States, species richness, the relative abundance of mice, and the number of nymphs were important variables in predicting the number of infected nymphs (Table 9). The number of infected nymphs increased with increasing deer abundance and an increasing number of nymphs, but was lower with increasing distance to the United States, greater species richness, and a higher relative abundance of mice (Fig. 6). The interaction between species richness and the relative abundance of mice (Fig. 7) and percent canopy cover received little support (Table 9). In addition, the overall abundance of small mammals was not correlated with the number of infected nymphs (rho = 0.028, p = 0.805).

**Table 8:** 95% confidence set of best ranked linear mixed models selected through all subsets regression analysis to explain the number of *Ixodes scapularis* nymphs infected with *Borrelia burgdorferi* (NIN)^a^ at various locations in the Thousand Islands in 2009 and 2010.

Model rank	Model terms	Parameters	R^2^	AIC_c_	Δ*_i_*	w*_i_*	Cumulative w*_i_*
1	Nymphs+Deer+Mice+Richness+Distance	8	0.45	126.86	0.00	0.2425	0.2425
2	Nymphs+Deer+Mice+Richness+Canopy+Distance	9	0.44	128.73	1.88	0.0949	0.3374
3	Nymphs+Deer+Richness+Distance	7	0.43	128.77	1.91	0.0934	0.4308
4	Deer+Mice+Richness+Distance	7	0.41	128.83	1.98	0.0903	0.5211
5	Nymphs+Deer+Mice+Distance+Richness*Mice	9	0.40	129.26	2.40	0.0731	0.5942
6	Deer+Mice+Richness+Canopy+Distance	8	0.42	129.62	2.76	0.0609	0.6551
7	Deer+Mice+Distance+Richness*Mice	8	0.34	130.29	3.44	0.0435	0.6986
8	Nymphs+Deer	5	0.40	130.62	3.76	0.0369	0.7355
9	Nymphs+Deer+Mice	6	0.41	130.83	3.97	0.0333	0.7688
10	Nymphs+Deer+Richness+Canopy+Distance	8	0.42	131.22	4.36	0.0274	0.7963
11	Nymphs+Deer+Richness+Canopy+Distance+Richness*Mice	10	0.42	131.34	4.48	0.0258	0.8221
12	Nymphs+Deer+Mice+Distance	7	0.41	131.37	4.51	0.0255	0.8475
13	Nymphs+Deer+Distance	6	0.40	131.76	4.90	0.0209	0.8684
14	Deer+Mice+Richness+Canopy+Distance+Richness*Mice	9	0.41	131.87	5.01	0.0198	0.8882
15	Nymphs+Deer+Mice+Canopy	7	0.40	132.53	5.67	0.0142	0.9024
16	Deer+Richness+Distance	6	0.38	132.55	5.69	0.0141	0.9165
17	Nymphs+Deer+Richness	6	0.39	132.81	5.95	0.0124	0.9289
18	Nymphs+Deer+Canopy	6	0.39	132.95	6.09	0.0116	0.9405
19	Nymphs+Deer+Mice+Richness	7	0.40	133.13	6.27	0.0105	0.9510

**Model** **terms:** Number of nymphs = nymphs caught per 2 person hours of dragging; Deer = average number of pellet groups per hectare; Mice = relative abundance of *Peromyscus leucopus*; Richness = small mammal species richness; Canopy = percent canopy cover; Distance = distance from United States mainland (km); Richness*Mice = interaction between Mice and Richness. Month was included as a random effect.

**Other abbreviations:** R^2^ = proportion of variance explained by the model, adjusted for number of terms and sample size; AIC_c_ = Akaike’s information criterion corrected for sample size; Δ*_i_* = difference in AIC_c_ from top-ranked model; w*_i_* = AIC weight [63].

^a^ Results are for transformed data (log(NIN+1)).

**Table 9:** Model-averaged parameter estimates and standard errors based on the 95% confidence set for variables potentially affecting the number of infected nymphal *Ixodes scapularis* (NIN)* collected at various locations in the Thousand Islands in 2009 and 2010.

			95% confidence interval	
Parameter	Estimate	Standard error	Upper	Lower
Deer	0.30	0.08	0.46	0.14
Distance	−0.29	0.11	−0.07	−0.50
Richness	−0.28	0.11	−0.07	−0.49
Mice	−0.15	0.07	−0.01	−0.30
Number of nymphs	0.15	0.07	0.29	0.02
Canopy	0.05	0.07	0.19	−0.08
Mice*Richness	−0.06	0.13	0.19	−0.32

Results are for transformed data (log(NIN+1)).

**Model** **terms:** Number of nymphs = nymphs caught per 2 person hours of dragging; Deer = average number of pellet groups per hectare; Mice = relative abundance of *Peromyscus leucopus*; Richness = small mammal species richness; Canopy = percent canopy cover; Distance = distance from United States mainland (km); Mice*Richness = interaction between Mice and Richness. Month was included as a random effect.

## Discussion

Deer abundance, distance to the United States, small mammal species richness, relative mouse abundance, canopy cover, and air temperature were all associated with the complex *B. burgdorferi* transmission cycle in the Thousand Islands. The structure of the small mammal community was an important factor in modulating the effects of species richness on the number of nymphs and the prevalence of *B. burgdorferi* infection: depending on the relative abundance of mice, high biodiversity did not always reduce the factors that contribute to the measurement of human disease risk. Dilution was evident in the effect of small mammal species richness on the overall number of infected nymphs.

### Geography, Temperature, Deer and Canopy Cover

Geography played an important role in Lyme disease risk in the Thousand Islands. Distance to the United States was an important predictor and negatively correlated with NON, NIP, and NIN. This is consistent with previous studies that report blacklegged ticks and *B. burgdorferi* are expanding from foci in the northeastern and north-central United States through the movement of migratory birds, deer, and possibly small mammal hosts [46]–[48], [67], [68]. Long-term monitoring in the Thousand Islands region could help to track the spread of ticks and *B. burgdorferi* and allow an assessment of changing disease risk. The pattern of fewer ticks farther from the United States suggests an expansion front that could be studied to learn more about the mechanisms of tick and *B. burgdorferi* establishment [47], [48].

The positive effect of climate warming on expansion of tick populations was supported by our results, with higher average daily minimum summer temperature at ground level positively correlated with tick abundance. The topography and geography of the islands and mainland sites created varied microclimatic conditions and the mean daily minimum temperature in the summer months ranked as an important predictor of the number of nymphs. The moderating effect of the St. Lawrence River may have played a role in this pattern, as mean daily minimum temperature in the summer was also negatively correlated with island size, and the three mainland sites had the lowest mean temperatures [69]. Island size may therefore also explain the pattern we saw, but size effects could not be teased out in our study. If island size is affecting tick populations, we would expect smaller islands to have higher densities of tick hosts than larger islands or the mainland because of less predation on small mammal hosts, less competition among hosts, lack of opportunities for host emigration, and increased immigration rates [70]–[73]. Host populations did not appear to be negatively associated with island size, but other factors, such as limited area for dispersal of ticks on smaller islands, may explain the correlation between the number of nymphs and mean daily minimum summer temperature.

The inclusion of island and mainland sites in our study area provided an opportunity to explore the effects of deer populations on Lyme disease risk [6], [9]–[14]. Relative deer abundance ranked as the most important positive predictor of NIN, and was also an important factor in the NON models. This is consistent with observations on isolated islands and using deer exclosures, where deer numbers can be limited [10]–[12]. With both mainland sites more open to deer movement and islands that are more restricted, we have the opportunity in the future to study the effects of deer population control at semi-isolated island sites. Overpopulation of deer has been a recent problem at some locations in the Thousand Islands [74] and management actions that control deer populations, usually for the benefit of vegetation, may also reduce the human risk of encountering infected nymphal ticks [12].

The risk of encountering a nymphal tick can also be reduced by avoiding closed-canopy areas.

The positive correlation we found between canopy cover and the number of nymphs is consistent with previous studies; ticks are more likely to inhabit forested areas than open fields [51], [75], [76]. The effect of open areas was apparent even within forested areas with different degrees of canopy cover. These results have implications for public education and the planning and maintenance of recreational facilities. Awareness of the increased risk of contracting Lyme disease in closed-canopy forested areas can help to direct the creation and modification of campsites, trails and picnic sites in public areas.

### Number of Nymphs

Unexpectedly, as the number of nymphs increased, the overall prevalence of *B. burgdorferi* infection in the population of nymphs decreased. This outcome may be the result of the recent emergence of the Lyme disease cycle in the Thousand Islands: tick populations may become established at new sites before *B. burgdorferi*, diluting the overall prevalence of *B. burgdorferi* infection that is largely restricted to adventitious ticks arriving on migratory birds [46], [47]. Another explanation may be the effect of deer populations: high deer densities may increase the number of nymphs (by allowing more adults to survive and reproduce), while also decreasing nymphal infection prevalence (because nymphs feeding on reservoir-incompetent deer will not be infected by *Borrelia*) [77]. Despite this pattern, a higher number of nymphs was nonetheless associated with a higher absolute number of infected nymphs, the important factor for evaluating disease risk. This may be because the NIN models, which combine NON and NIP, are more strongly influenced by factors that affect the number of nymphs than factors that affect the prevalence of *B. burgdorferi* infection, as evidenced by the large amount of variation in the data explained by the NON models in comparison to the NIP models. Therefore, an increase in the number of nymphs overwhelmingly increases the odds of encountering an infected nymph, despite a decrease in the overall prevalence of infection. Several studies examining the factors that affect disease risk have focussed on the prevalence of *B. burgdorferi* infection in nymphs as the outcome variable, assuming a strong link to the number of infected nymphs and, therefore, human risk [16], [22], [28]. However, as has been seen in European studies [78], the factors that affect the number of infected nymphs and human risk in the Thousand Islands are more strongly linked to those factors that affect the overall number of nymphs than those that affect nymphal infection prevalence.

The small amount of variation in the data explained by the NIP models suggests that random variation or other factors that were not measured influence the prevalence of infection in nymphs. We did not examine the effects of the time since tick establishment [38], the presence of all potential hosts (i.e., squirrels, foxes, skunks, raccoons, birds), or the proportion of each host in the community. Thus, while the combined effects of species richness and the dominance of a highly competent reservoir host such as mice do seem to affect nymphal infection prevalence, this relationship alone provides an incomplete picture of what determines local infection rates in ticks.

### Biodiversity and Small Mammal Hosts

Higher species richness was associated with a decrease in human disease risk, as measured by the number of infected nymphs. Also consistent with the dilution theory, the prevalence of *B. burgdorferi* infection in nymphs was lower with increasing biodiversity when the relative abundance of mice was low. As more species are added, some of those new species are likely to be less competent hosts and because hosts that are not mice make up a large proportion of the community, fewer ticks become infected with *B. burgdorferi*. The dilution theory was also evident when examining the effects of biodiversity on the number of nymphs. When mice comprised more than half of the small mammal community, higher species richness was correlated with lower nymph abundance. This may be because the other small mammals that comprised the species-rich communities where mice dominated were poor quality hosts, removing and eating a large proportion of feeding ticks through grooming [25]. Alternatively, the overall effect of other good quality hosts, such as shrews [17], may have been reduced when mice dominated. This limiting of the effect of other competent and good quality hosts would also explain the negative effect of mouse abundance on the number of infected nymphs. The importance of distance to the United States, which was negatively correlated with species richness, may also be contributing to the dilution effects we observed. A full survey of all tick hosts might help to elucidate the mechanisms behind the observed effects of small mammal species richness on Lyme disease risk in different small mammal communities.

We also found evidence for a context-dependent role of biodiversity, and situations that were not consistent with the dilution theory. The structure of the small mammal community, specifically the relative abundance of mice, modulated the effects of biodiversity on the number of nymphs and the prevalence of *B. burgdorferi* infection in nymphs. Species richness had little effect on the prevalence of infection in ticks at sites with a high relative abundance of mice. This could be because mice are very competent hosts [15]–[17] and when there are many of them relative to other small mammals, they can maintain a high level of infection in the tick population. This is a unique finding; other studies that have looked at the effects of species richness have assumed that higher species richness is correlated with a lower proportion of mice and that mice dominate communities with low species richness [16], [26]. However, these assumptions were not true at all of our sites and, when considered independently, the relative abundance of mice was negatively correlated with the number of infected nymphs. In addition, when the relative abundance of mice was low, higher species richness had little effect on the number of nymphal ticks, perhaps because of the positive correlation between species richness and the overall abundance of small mammals. That is, a greater number of animals (regardless of species) increases the chance that a tick will find a host and survive. While the overall effect of species richness on the NON, NIP, and NIN was negative, our results suggest that the degree of risk is dependent on the composition of the small mammal community in some situations. Our focus on the small mammal community included the most competent reservoirs of *B. burgdorferi* [16], [19], [21] and was logistically feasible. However, a complete survey of the presence and proportion of all potential hosts, including birds, large mammals, meso-mammals, and reptiles may provide an even better picture of the role of biodiversity in the Lyme disease cycle. Theoretical models have shown that both dilution and amplification of Lyme disease risk are possible depending on the abundance, competence, and quality of different hosts, and recent empirical studies have questioned the assertion that increasing biodiversity reduces human disease risk [31], [79], [80].

## Conclusion

Our study is one of the few to evaluate the predictions of theoretical models that explore dilution and amplification effects in the Lyme disease system in eastern North America [28], [31], [79]. Higher small mammal species richness was associated with fewer infected nymphs and, therefore, decreased human disease risk. However, we also found that the relative abundance of mice is an important factor in modulating the effects of species richness on human disease risk factors: high biodiversity does not always reduce nymphal infection prevalence or the number of nymphs. Our study supports the assertion that community composition may be more important in understanding human disease risk than standard measures of biodiversity such as species richness [29]. In addition to insight on the factors affecting the distribution of ticks and *B. burgdorferi* in a zone of Lyme disease emergence in eastern North America, these results contribute empirical evidence to the debate about the role of biodiversity in reducing human disease risk worldwide.
